# Effect of Stereochemical Configuration on the Transport and Metabolism of Catechins from Green Tea across Caco-2 Monolayers

**DOI:** 10.3390/molecules24061185

**Published:** 2019-03-26

**Authors:** Zeyi Ai, Shuyuan Liu, Fengfeng Qu, Haojie Zhang, Yuqiong Chen, Dejiang Ni

**Affiliations:** Key Laboratory of Horticultural Plant Biology, Ministry of Education, College of Horticulture and Forestry Sciences, Huazhong Agricultural University, Wuhan 430070, China; aizeyi@webmail.hzau.edu.cn (Z.A.); lsy299792458@126.com (S.L.); quff@webmail.hzau.edu.cn (F.Q.); zhjie13653809169@163.com (H.Z.); chenyq@mail.hzau.edu.cn (Y.C.)

**Keywords:** stereochemical configuration, cis–trans catechins, bidirectional transport, efflux pumps, metabolism

## Abstract

The transcellular transport and metabolism of eight green tea catechins (GTCs) were studied in Caco-2 monolayers, with the aim of investigating the effect of cis–trans isomerism on the membrane permeability and biotransformation of GTCs. The results showed that the catechin stereochemistry significantly affects the efflux transport rather than the absorption transport in the Caco-2 monolayers. The trans catechins showed a better transcellular permeability than their corresponding cis (epi) catechins in the efflux transport, as the efflux amount of trans catechins were all significantly higher than that of the cis (epi) catechins at each concentration and each time point tested. Moreover, the relative contents of the (+)-catechin (C)-*O*-sulfate, (+)-gallocatechin (GC)-*O*-sulfate, (−)-catechin gallate (CG)-*O*-sulfate, and (−)-gallocatechin gallate (GCG)-*O*-sulfate in the efflux transport were 2.67, 16.08, 50.48, and 31.54 times higher than that of the (−)-epicatechin (EC)-*O*-sulfate, (−)-epigallocatechin (EGC)-*O*-sulfate, (−)-epicatechin gallate (ECG)-*O*-sulfate, and (−)-epigallocatechin gallate (EGCG)-*O*-sulfate, respectively. It indicated that more metabolites were observed after the transcellular efflux of trans catechins. Furthermore, after two hours of incubation, the GTCs could significantly increase the expression of multidrug resistance-associated protein 2 (MRP2) and breast cancer-resistance protein (BCRP), and decrease the expression of *P*-glycoprotein in the Caco-2 cells. The regulation of GTCs on P-glycoprotein, MRP2, and BCRP could also be significantly influenced by the chemical and dimensional structure. In a conclusion, catechin stereochemistry significantly affects the transport and metabolism of GTCs when refluxed in the Caco-2 monolayers.

## 1. Introduction

**Figure 1:**
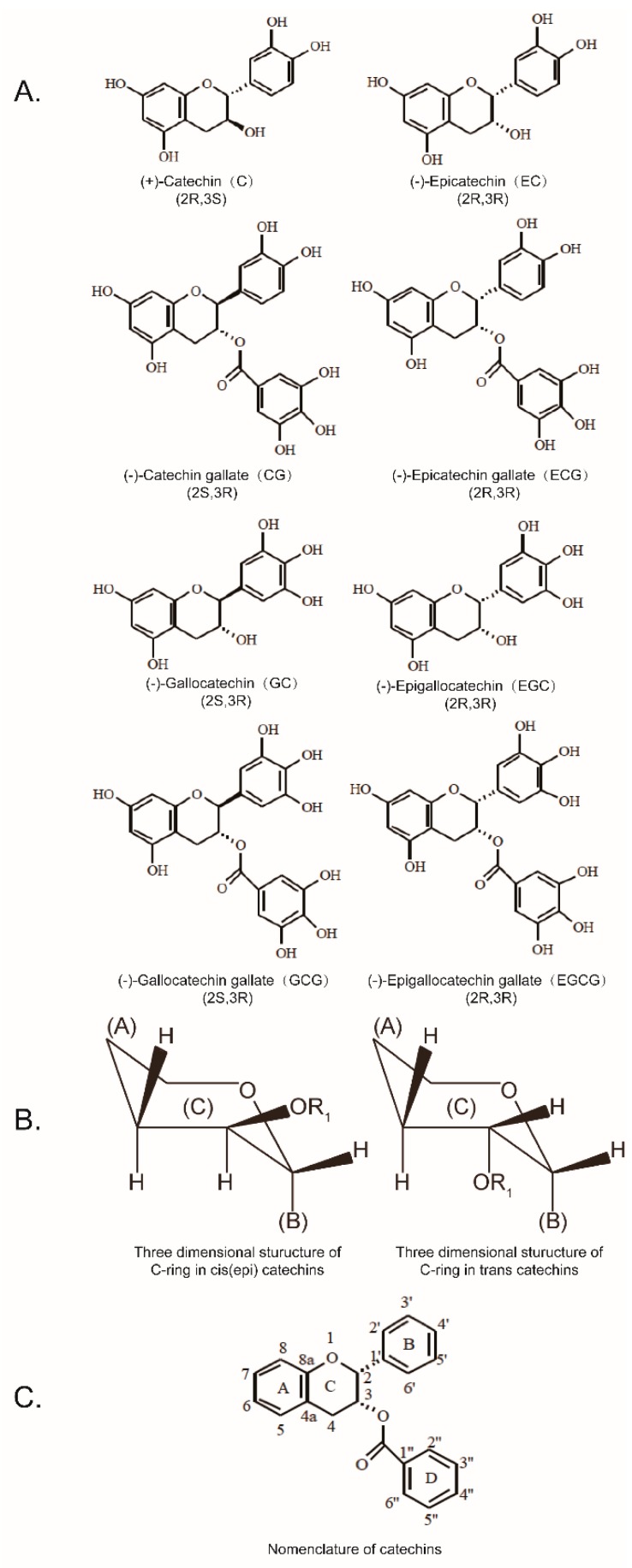
Eight catechins (**A**); three dimensional structure of the C-ring between the cis–trans catechins (**B**); nomenclature of catechins (**C**).

Green tea catechins (GTCs), the major beneficial components in green tea, include eight native monomeric catechins, which are (−)-epicatechin (EC), (+)-catechin (C), (−)-epigallocatechin (EGC), (+)-gallocatechin (GC), (−)-epicatechin gallate (ECG), (−)-catechin gallate (CG), (−)-epigallocatechin gallate (EGCG), and (−)-gallocatechin gallate (GCG) [1]. C, CG, GC, and GCG are the corresponding cis–trans isomers of EC, ECG, EGC, and EGCG. Their chemical structures are shown in Figure 1. Cis–trans isomers are stereoisomers, which are a pair of molecules with the same formula, although their functional groups rotate into a different orientation in three-dimensional space. As a result of the same molecular formula and functional groups, cis and trans isomers often have the same chemical properties. However, the different orientation of the functional groups often leads to different physical properties and biological activity.

The beneficial effects of GTCs on human health are of great interest to scientists. A series of pharmacological effects of GTCs have been reported, including anti-oxidative activity, inhibition of AP-1 transactivation, inhibition of the proteasome activity, inhibition of epidermal growth factor receptor (EGFR), and other growth factor signals [2,3,4,5,6]. All of these potential bioactivities and their overall significance in disease prevention are dependent on the absorption, distribution, metabolism, and excretion (ADME) properties of GTCs within the body after ingestion, and the reducing properties of the resulting metabolites, so it is of great importance to understand the biotransformation and bioavailability of GTCs. For oral agents like GTCs, bioavailability reflects the rate and extent of gastrointestinal (GI) tract absorption. The oral bioavailability, the fraction of an oral administered drug that reaches the systemic circulation, depends on the transcellular uptake in humans. The oral bioavailability of cis (epi) catechins (EC, EGC, ECG, and EGCG) has been demonstrated to be low in Caco-2 cells [7], rats [8,9], and humans [10,11]. With the metabolites of cis (epi) catechins in Caco-2 cells, urine, bile, and plasma being studied [8,12,13,14], it still lacks the identification of the metabolites of trans GTCs. Moreover, the transcellular transport and metabolism of naturally occurring trans catechins (C, CG, GC, and GCG) is never elucidated. To have a more comprehensive understanding of the GTCs, a thorough investigation on the transport and metabolism of trans catechins is necessary.

Multidrug resistance-associated protein 2 (MRP2; ABCC2); breast cancer resistance protein (BCRP; ABCG2); and P-glycoprotein, encoded by the human multidrug resistance gene 1 (MDR1; ABCB1), are all members of the ATP-binding cassette (ABC) transporter family, and function as efflux pumps for endogenous and exogenous compounds [15]. It is reported that MK-571, an inhibitor of MRP2, BCRP, and *P*-glycoprotein [16], could inhibited the efflux of cis catechins. Further research confirmed that ECG and EGCG are substrates for MRP2 [17,18]. However, the role of MK-571 in affecting the cellular level of trans catechins has never being investigated. Furthermore, the information on the role of catechins in the expression of BCRP, *P*-glycoprotein, and MRP2 at a molecular level is really limited, and more research in this area is needed.

The human intestinal Caco-2 cell monolayer model has been generally accepted as an in vitro model, to rapidly assess the cellular permeability of potential drug candidates, to elucidate the pathways of drug transport, and to study the pre-systemic drug metabolism in the intestine [19]. Similar to the human intestine, Caco-2 cells also express transporters, such as MRP, BCRP, and *P*-glycoprotein [20]. In the work presented here, the Caco-2 model was used to study the transport and metabolism of eight GTCs in order to investigate the differences in the membrane permeability and biotransformation between cis–trans catechins in vitro. The gene and protein expression profiles of BCRP, *P*-glycoprotein, and MRP2 were also examined so as to evaluate the effect of the tea catechins on them at a molecular level. This research explains the transport mechanism of the trans catechins and provides insight into the processes that give rise to potential bioactive forms in the small intestine.

## 2. Results and Discussion

### 2.1. Bidirectional Transport of Eight GTCs

When eight GTCs (300 μmol/L) were loaded onto the apical chambers, no significant absorption transport was observed, as the *P_app_* values were much less than 1 × 10^−6^ cm/s (Table 1). It is well known that the absorption of oral administration in vivo has a close correlation between the permeability across the Caco-2 cell monolayers [21]. Therefore, cis (epi) and trans GTCs with *P_app_* values less than 1 × 10^−6^ cm/s in the Caco-2 cell monolayers may all have a poor absorption (<30%) in humans. The presented investigations were consistent with the a previous study on the cis (epi) catechins (EC, EGC, ECG, and EGCG) in the Caco-2 cells [10]. As the oral bioavailability is the fraction of an oral administered drug that reaches systemic circulation, we predicted that the GTCs all had a very low oral bioavailability in humans. Also, in vivo experiments showed that the low bioavailability of green tea in animals makes itself much less available for its bioactivities [22]. The plasma concentration in rats following oral administration has been reported as being as high as 53 uM at a dose of 250 mg/kg for (−)-EC, 33.5 uM at a dose of 650 mg/kg for ECG, and 244.3 uM at a dose of 2500 mg/kg for EGCG [9], respectively. For the intragastric administration, the bioavailability was 31% for (−)-EC, 13.7% for EGC, and 0.1% for EGCG in rats [23]. Although no absolute bioavailability has been reported in humans, the low absorption of GTCs also suggests a low bioavailability for its bioactivities.

As shown in Table 1, the transport of GTCs from BL to AP was more extensive compared with the transport from AP to BL. MK-571 could significantly inhibit the efflux of eight catechins, and increase the transepithelial absorption of the catechins, which led to a decrease in the efflux ratios (less than 1.5). It indicated that active transporters like BCRP, *P*-glycoprotein, and MRP2 may be involved in the efflux of GTCs, as they could all be inhibited by MK-571 in previous reports [16]. These results were consistent with the work reported by Zhang et al. [7], who found that the high efflux of EC and EGC can be inhibited by MK-571 during secretion in Caco-2 cells. To our knowledge, this is the first report establishing that trans catechins (C, GC, CG, and GCG) have limited transepithelial absorptions with low *P_app_* values, and a significant efflux inhibited by MK-571 during secretion. Moreover, GTCs, as the candidates of BCRP substrates, might compete with other substrates for BCRP in Caco-2 cells, which may provide an explanation for GTCs being able to increase the cellular accumulation and inhibit efflux transport of mitoxantrone (a substrate of BCRP) in Caco-2 monolayers, as previously reported [24].

Although no significant differences were found in the absorption transport between cis (epi) and trans catechins, the *P_app_* values in the efflux transport of trans catechins (C, CG, and GC) were significantly higher than the values of their corresponding cis (epi) catechins (EC, ECG, EGC) except GCG and EGCG. Moreover, the efflux ratios of the trans catechins were also higher than the values of the cis (epi) catechins. Taken together, the cis–trans isomerism may affect the permeability of the catechins through the lipid bilayer.

**Figure 2:**
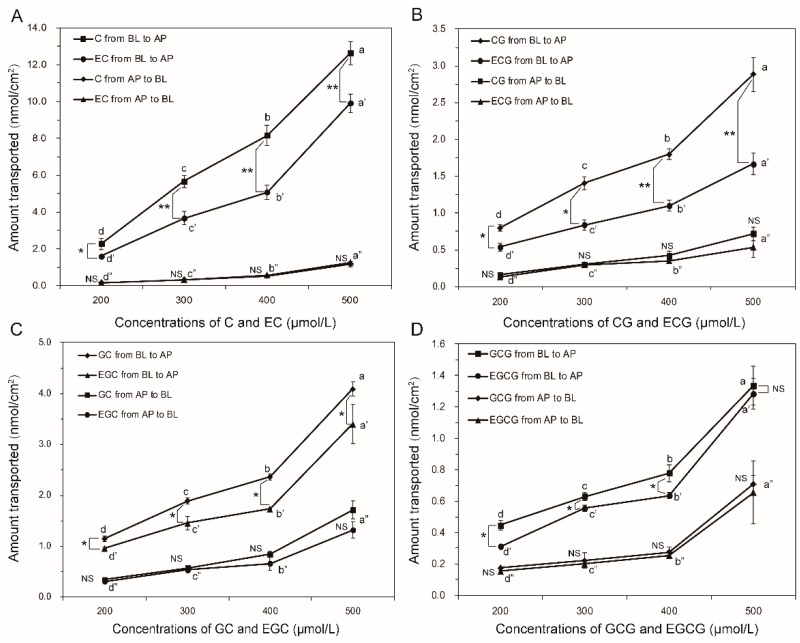
Effects of concentration on the transport of trans catechins and cis (epi) catechins (**A**) C and EC, (**B**) CG and ECG, (**C**) GC and EGC, and (**D**) GCG and EGCG across Caco-2 cell monolayers in both directions. The apical (AP) to basolateral (BL) fluxes at concentrations from 200 μM to 400 μM are all linear for up to 2 h in a concentration-dependent manner, with relative independent *P_app_* values, respectively (*p* > 0.05), while the BL to AP fluxes are increased with the increasing concentrations, with a saturation at concentrations higher than 300 μM for the corresponding *P_app_*, which decreased with loading concentrations greater than 300 μM. Surges at 500 μmol/L were observed in the fluxes of eight catechins from both directions. Each value was the average of three different samples; the different lowercase letters in each line indicate a significant difference at a 0.05 level between the different concentrations; for cis–trans catechins, * and ** indicate significantly different at a 0.05 and 0.01 level respectively, While NS indicates not significant.

**Figure 3:**
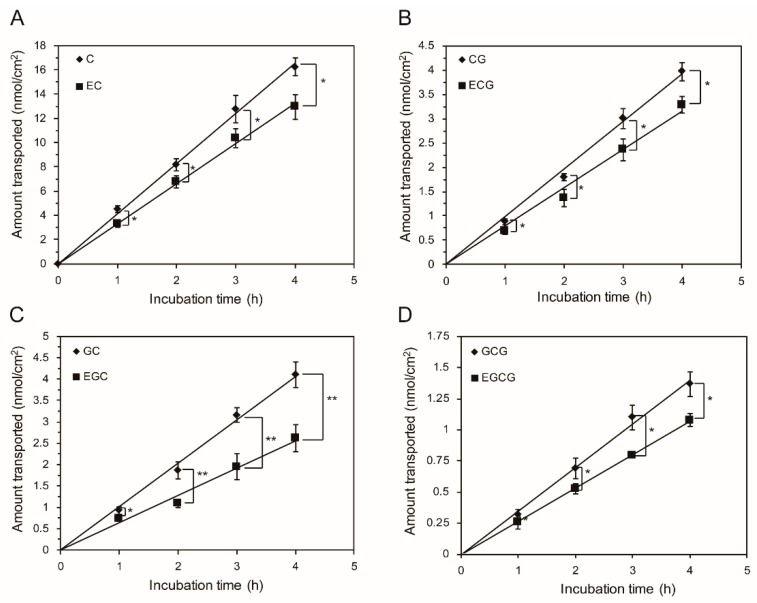
Effect of incubation the time on the efflux transport of trans catechins and cis (epi) catechins at 300 μM, across Caco-2 cell monolayers (**A**) C and EC, (**B**) CG and ECG, (**C**) GC and EGC, and (**D**) GCG and EGCG. The efflux amount of each catechin increased linearly with the incubation time. Each value was the average of three different samples; * and ** indicate significantly different at a 0.05 and 0.01 level between cis and trans catechins, respectively.

In this way, the effects of concentration (200 μmol/L to 500 μmol/L) on the transport of trans catechins and cis (epi) catechins in both directions were determined. As shown in Figure 2, the fluxes from both directions of GTCs were all increased in a concentration-dependent manner. Surges at 500 μmol/L were observed in the fluxes of eight GTCs from both directions. This might be associated with the integrity of the Caco-2 monolayers, as significantly decreased transepithelial electrical resistance (TEER) values (less than 400) were observed after loading the GTCs at 500 μmol/L, although a Cell Counting Kit-8 (CCK-8) test indicated that GTCs at 500 μmol/L were nontoxic to the cells (as shown in Figure S1). During the transport from apical (AP) to basolateral (BL), no significant differences between cis–trans catechins were found at each concentration. In contrast, the efflux amounts of C, CG, GC, and GCG were significantly higher than EC, ECG, EGC, and EGCG at each concentration (200μmol/L to 400 μmol/L). Moreover, the efflux amounts of the trans catechins (C, CG, GC, and GCG) were higher than that of their corresponding cis (epi) catechins (EC, EGC, ECG, and EGCG) at each time point tested (Figure 3). Taken together, the results of absorption and secretion transport present data that indicates that the catechin stereochemistry could significantly affects the efflux transport rather than the absorption transport in the Caco-2 cell monolayers. Considering the involvement of the efflux pumps in secretion, we hypothesized that more trans catechins were secreted back into the intestinal lumen by efflux pumps than cis (epi) catechins, after same amount cis and trans catechins were absorbed from intestine, which would lead to a higher oral bioavailability of cis (epi) catechins in humans. This could provide a reason to explain why (−)-epicatechin had a better oral bioavailability than (+)-catechins in humans, as previously reported [25]. In summary, stereochemical characteristics could be a crucial factor with regard to catechins absorption in humans.

### 2.2. Metabolites of Eight GTCs

In order to study the metabolites of the eight GTCs, the deprotonated molecular ions of eight GTCs and their possible metabolites were targeted by mass spectrometry with electrospray ionization in a negative mode in the transport experiment, from both directions. Further MS/MS analysis confirmed that the peaks were the metabolites of th ecatechins (as shown in Figures S2–S5, respectively). As most of the conjugated catechins are commercially unavailable, the conjugated metabolites were quantified as equivalents to their available free-form standards, relative to the internal standard (ethyl gallate) [26].

In transporting BL to AP across Caco-2 monolayers (Table 2), sulfate and methylated conjugation represents the major metabolic pathways for each GTC. This was consistent with the previous reports of cis (epi) catechins. Early reports showed that sulfate conjugate seemed to be the major metabolites of catechins in humans, while glucuronide was the major metabolites of catechins in rats [27,28]. However, Kayleigh et al. reported EC-glucuronide and EGC-glucuronide in urine after drinking green tea, with no glucuronidation occurring in ECG or EGCG [26]. Ottaviani et al. confirmed that the glucuronide in the non-methylated forms were also the main metabolites of EC in human plasma and urine [29]. These reports revealed that glucosidinization is also an important metabolic pathway of non-gallated catechins in humans. However, none of the glucuronidated metabolites were observed for any of the eight GTCs in the presented work, although the Caco-2 cell originated from humans. This may be the result of the differences between the pre-systemic metabolism experiments in vitro and the systemic metabolism experiments in vivo. Li Zhang and colleagues [7] also found a methyl-sulfate conjugate in the cis (epi) catechins metabolites in the transport experiments in the Caco-2 cells. However, in the work presented here, the methyl-sulfate conjugate was only observed for GC, EGC, CG, and GCG, which may be on account of the limited transport time (2 h). Quantitative analysis showed that the relative contents of the trans catechins metabolites (except *O*-me-GC) were all significantly higher than that of their corresponding cis (epi) catechins. The relative contents of the C-*O*-sulfate, GC-*O*-sulfate, CG-*O*-sulfate, and GCG-*O*-sulfate were 2.67, 16.08, 50.48, and 31.54 times higher than that of the EC-*O*-sulfate, GC-*O*-sulfate, ECG-*O*-sulfate, and EGCG-*O*-sulfate, respectively. The methylated metabolites of the GTCs presented a similar tendency, except for *O*-me-GC and *O*-me-EGC. We found that the total content of the *O*-me-GC (18.54 ± 1.74 nmol) was significantly lower than the *O*-me-EGC (30.10 ± 3.35 nmol), but the content of the GC-*O*sulfate (510.85 ± 61.72 nmol) was significantly higher than the EGC-*O*sulfate (31.77 ± 1.97 nmol). Therefore, we hypothesized that the most GCs were transformed into a GC sulfate conjugate after loading a finite concentration, which would lead to a GC shortage for methylation. As more metabolites were observed quantitatively in the trans catechins when compared with their corresponding cis (epi) catechins after transport from BL to AP in Caco-2 monolayers, we speculated that more metabolites of trans catechins were fluxed to lumen after absorption than cis (epi) catechins in humans. With the higher BL to AP transport in the transcellular transport assay, the relative content data also indicated that the trans catechins could exist (quantitatively) in the intracellular space of the Caco-2 cells than the cis-isomers, and this leads to an increasing possibility that the cytosolic phase II enzymes could associate with their substrates during incubation.

**Figure 4:**
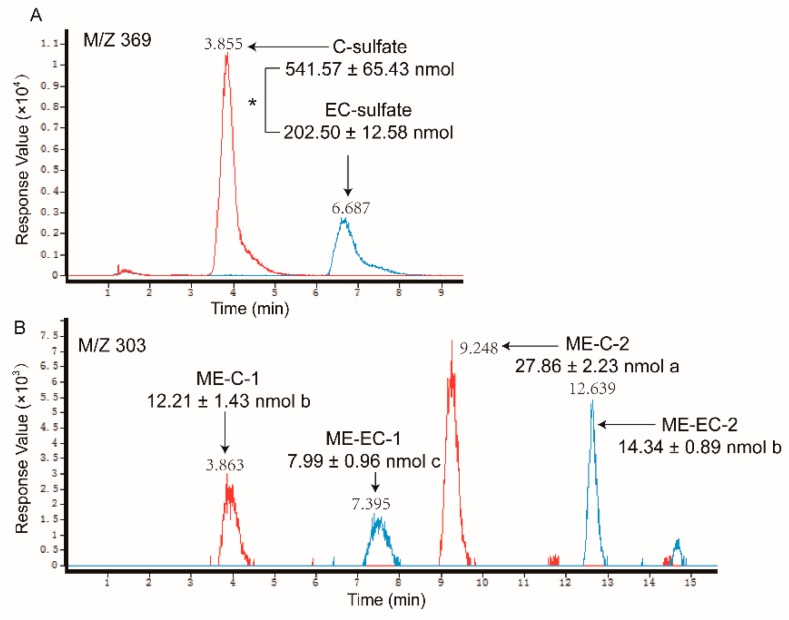
LC chromatograms of the samples taken from apical sides after loading C (line in red) and EC (line in blue) in the basolateral side, obtained by negative electrospray ionization (ESI)/MS interface extracted with (**A**) 369 [M − H]^−^ and (**B**) 303 [M − H]^−^. The relative content of the catechin metabolites were measured as free-form equivalents, relative to the internal standard. Each value was the average of three different samples; * indicates significantly different at a 0.05 level. The mean values with the same lowercase letters indicate no significant difference at a 0.05 level.

**Figure 5:**
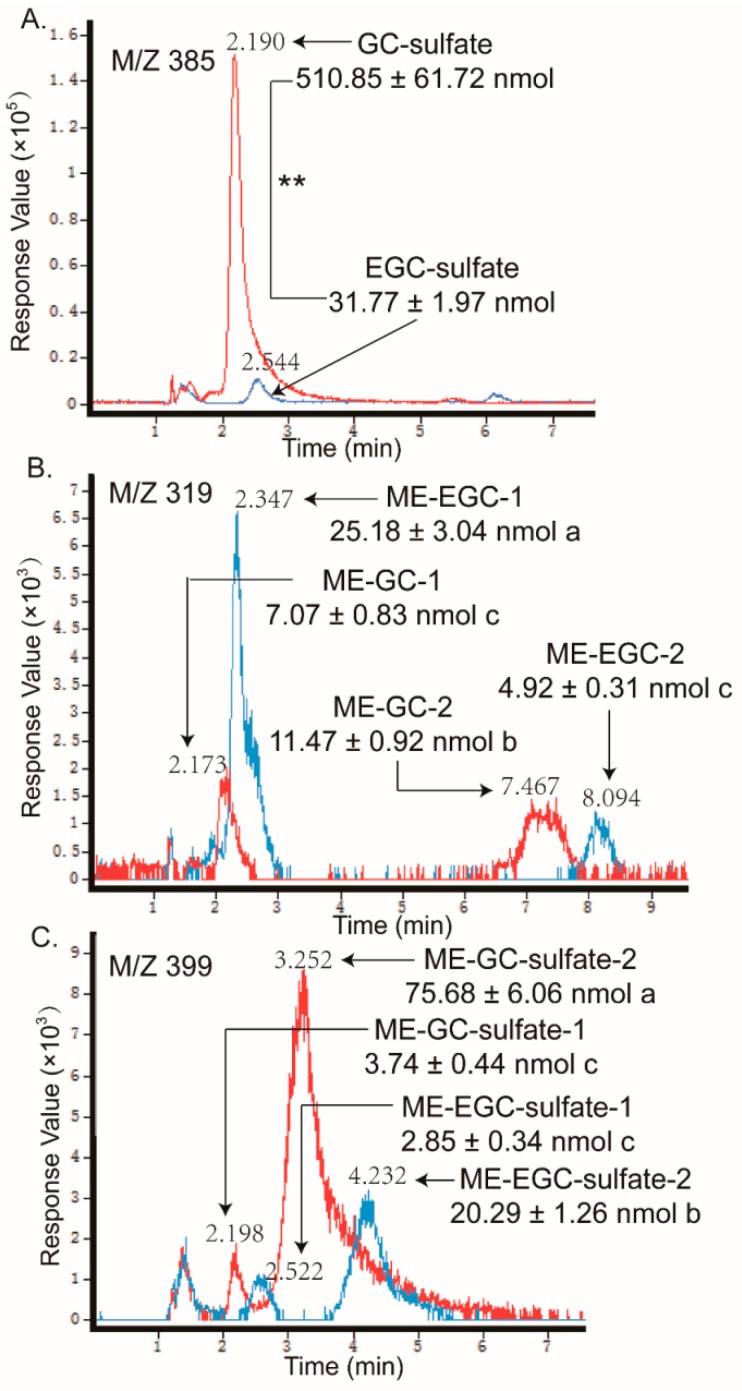
LC chromatograms of the samples taken from apical sides after loading GC (line in red) and EGC (line in blue) in the basolateral side, obtained by negative ESI/MS interface extracted with (**A**) 385 [M − H]^−^, (**B**) 319 [M − H]^−^, and (**C**) 399 [M − H]^−^. The relative content of the catechin metabolites were measured as free-form equivalents, relative to the internal standard. Each value was the average of three different samples; ** indicates significantly different at a 0.01 level. The mean values with the same lowercase letters indicate no significant difference at a 0.05 level.

**Figure 6:**
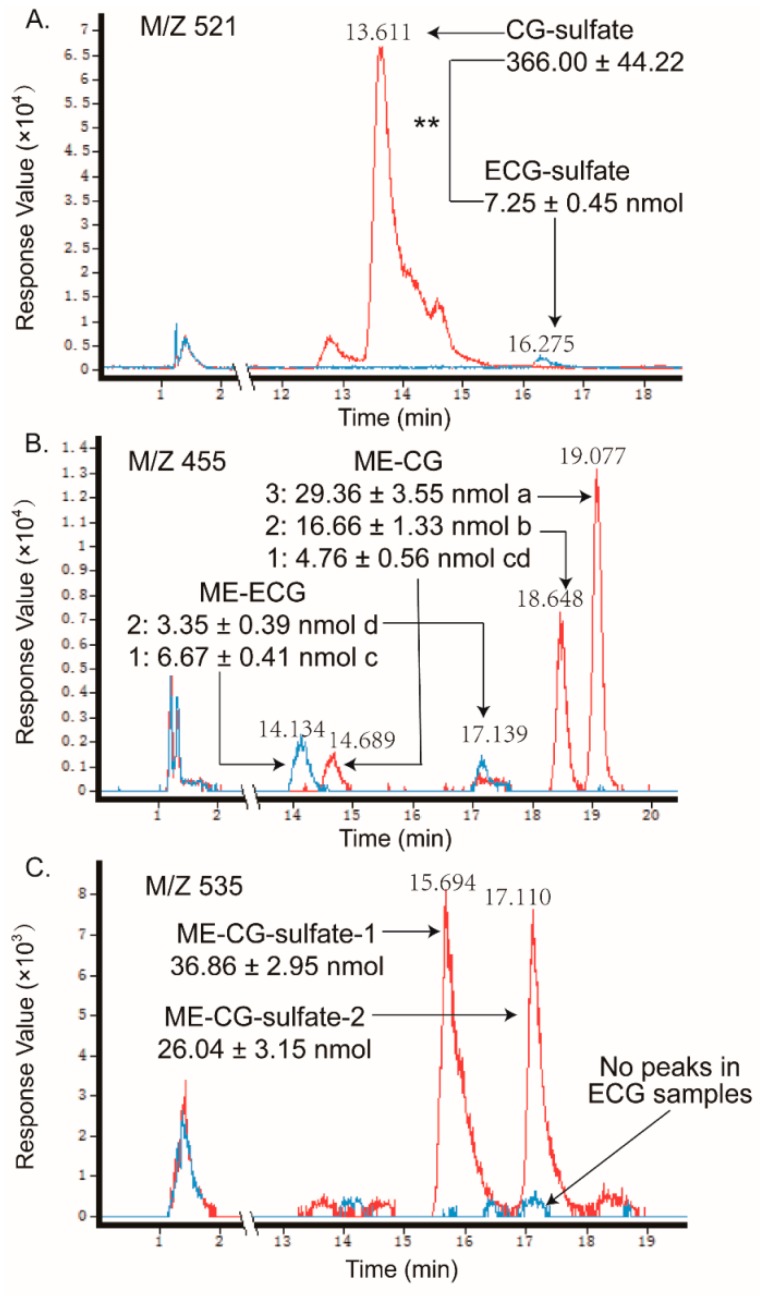
LC chromatograms of the samples taken from apical sides after loading CG (line in red) and ECG (line in blue) in the basolateral side, obtained by the negative ESI/MS interface extracted with (**A**) 521 [M − H]^−^, (**B**) 455 [M − H]^−^, and (**C**) 535 [M − H]^−^. The relative content of the catechin metabolites were measured as free-form equivalents, relative to the internal standard. Each value was the average of three different samples; ** indicates significantly different at a 0.01 level. Mean values with the same lowercase letters indicate no significant difference at a 0.05 level.

**Figure 7:**
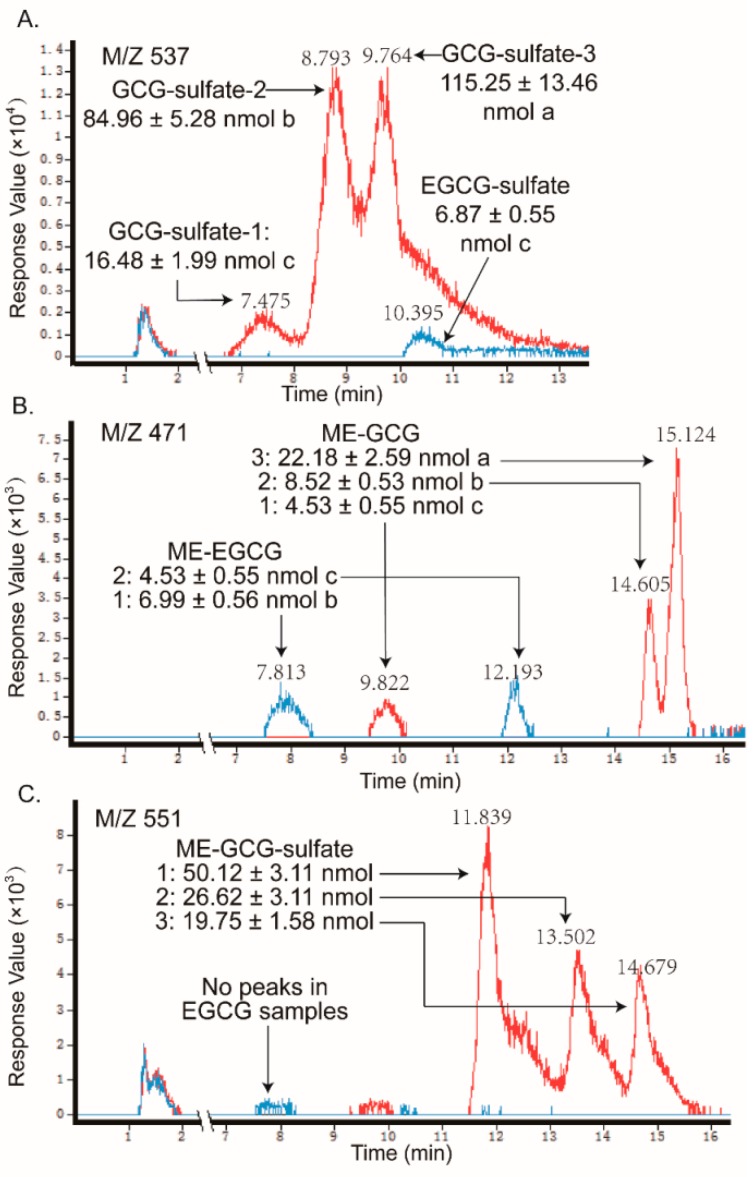
LC chromatograms of the samples taken from apical sides after loading GCG (line in red) and EGCG (line in blue) in the basolateral side, obtained by negative ESI/MS interface extracted with (**A**) 537 [M − H]^−^, (**B**) 471 [M − H]^−^, and (**C**) 551 [M − H]^−^. The relative content of the catechin metabolites were measured as free-form equivalents, relative to the internal standard. Each value was the average of three different samples; Mean values with the same lowercase letters indicate no significant difference at a 0.05 level.

As shown in Figure 4 and Figure 5, as the sulfate conjugates of the non-gallated catechins (C, EC, GC, and EGC) each got one peak and the methylations of the non-gallated catechins each got two peaks, respectively, conjugations in the non-gallated catechins seemed to take place at the same position for the cis–trans isomers. Methylation tends to take place at the 3′-OH or 4′-OH position of C and EC [27,30]. The methyl group was also found to conjugate at the 4′-OH of EGC [13,31]. Similarity, two peaks were observed in the *O*-me-GC-*O*-sulfate and *O*-me-EGC-*O*-sulfate, respectively. However, none of the methylated sulfate conjugates were found in both of the C and EC samples. As for the gallated catechins (Figure 6 and Figure 7), the methylation of CG and GCG each had three peaks, while the methylation of ECG and EGCG each had two peaks, implying that CG and GCG each had one more conjugation position than ECG and EGCG when methylated. As the methylations of ECG and EGCG at 4′-OH and 4″-OH were previously reported [12,32], further analysis is required in order to identify the conjugation position of the methyl group in CG and GCG. Moreover, methylated sulfate conjugates were only observed in the CG and GCG samples, while none were detected in the samples treated with ECG and EGCG. So, more types of metabolites were found qualitatively in the trans catechins in the gallated form when compared with their corresponding cis (epi) catechins after transport from BL to AP.

**Figure 8:**
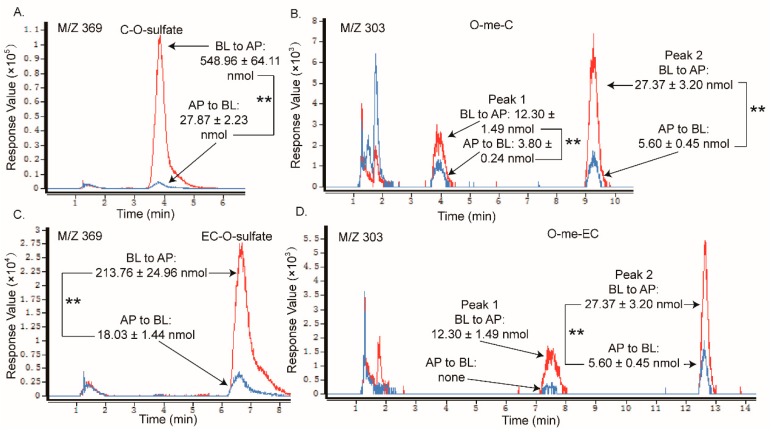
LC chromatograms of the samples taken from apical sides (line in red) and from the basolateral side (line in blue) after loading C and EC in the corresponding donor side, obtained by negative ESI/MS interface extracted with *m*/*z* (**A**) 369 [M − H]^−^, (**B**) 303 [M − H]^−^, (**C**) 369 [M − H]^−^, and (**D**) 303 [M − H]^−^. Each value was the average of three different samples; ** indicates significantly different at a 0.01 level.

In addition, the samples in the receiver chambers of the AP to BL transport with non-gallated catechins (C, EC, GC, and EGC) showed that the same types of metabolites formed as that in the receiver chambers of the BL to AP transport, but with much fewer amounts than the later one (like C and EC in Figure 8). What is more, in the absorption transport of the gallated catechins, none of the sulfate conjugation or methylation metabolites were found in the receiver chamber. It suggested that the sulfated and methylated metabolites might also have efflux transporters involved, especially for the gallated catechins. As the anionic nature is an important characteristic for the MRP2 substrates, sulfated and methylated metabolites might also be transported by MRP2 in the Caco-2 cells. The efflux and extensive metabolism of the GTCs makes them much less available for their bioactivities, which leads to a very low bioavailability, especially for the gallated catechins.

### 2.3. Effect of Eight GTCs on the Expression of MRP2, BCRP, and P-glycoprotein in Caco-2 Cells

As little is known about the effect of the stereoisomer-specific differences in the previously observed catechins-induced effect on the efflux transporters in humanw [29], the gene and protein expression profiles of MRP2, *P*-glycoprotein, and BCRP were investigated using qRT-PCR and Western blotting. Firstly, the mRNA levels of *P*-glycoprotein were significantly inhibited after treating with eight catechins (300 μmol/L) for 2 h (Figure 4A). Multiple comparisons showed that the chemical and dimensional structure of the catechin could significantly influence the downregulation of the *P*-glycoprotein at a transcription level, and at least two preliminary results could be drafted—one is a galloyl moiety enhancing the downregulation of P-glycoprotein, with the gallated catechin (i.e., EGCG) having a better inhibitory effect than the non-gallated one (i.e., EGC); the other is cis–trans isomerization enhancing the downregulation of *P*-glycoprotein, with the cis-catechin (i.e., EGCG) having a better inhibitory effect than the trans-catechin (i.e., GCG). As for the protein level of P-glycoprotein, the GTCs significantly decreased the expression of *P*-glycoprotein, except for C and EC. These results may give a reason as to why EGC, ECG, and EGCG (except EC) could all increase the cellular accumulation of the *P*-glycoprotein substrates, rhodamine-123, and daunorubicin in the KB-C2 cells, as previously reported [33]. Therefore, we speculated that the inhibitory effect of GTCs on the *P*-glycoprotein function was achieved by decreasing the protein expression of *P*-glycoprotein. However, the inhibitory effect of EGCG on *P*-glycoprotein at a transcription and translation level were contrary to a previous study, in which EGCG increased the mRNA and protein levels of *P*-glycoprotein in the Caco-2 cells. This opposite result can be caused by the different concentrations (300 μmol/L and 10 μmol/L) and different incubation time (2 h and four weeks) that were used.

**Figure 9:**
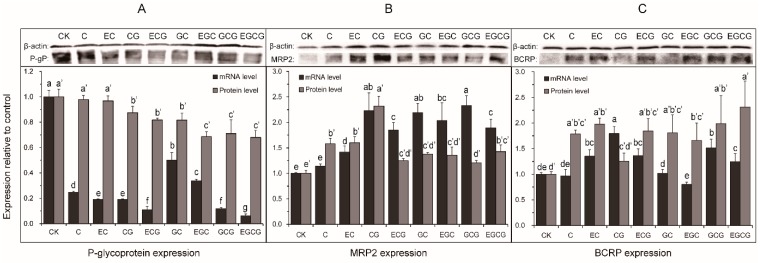
The mRNA level and protein expression of *P*-glycoprotein (**A**), MRP2 (**B**), and BCRP (**C**) in Caco-2 cells after treatment with C, EC, CG, ECG, GC, EGC, GCG, and EGCG for 2 h. Each value was the average of three different samples; mean values with the same lowercase letters indicate no significant difference at a 0.05 level.

Secondly, after treating with eight GTCs for 2 h, C, EC, CG, ECG, GC, EGC, GCG, and EGCG significantly increased the mRNA levels of MRP2 by 14.05%, 41.67%, 123.09%, 85.18%, 119.33%, 103.58%, 133.15%, and 89.57%, respectively (Figure 9B). As for the protein level, C, EC, CG, ECG, GC, EGC, GCG, and EGCG significantly increased MRP2 by 58.33%, 60.00%, 132.00%, 24.67%, 37.67%, 35.67%, 20.67%, and 42.67%, respectively. No apparent pattern was found between the cis–trans catechins, but the mRNA and protein level of the MRP2 treated with CG were both significantly higher than those treated with ECG. At a translational level, the inductive effect of EGCG on MRP2 was contrary to a previous study performed by Petra Hirsova [29], who considered that EGCG significantly decreased the protein level of MRP2 on the hepatocyte canalicular membrane in rats after 24 h of incubation. This opposite result can be caused by the different function of MRP2 in the different tissues from different species. The MRP2 expressed in the canalicular (apical) part of the hepatocyte are mainly responsible for biliary transport, while those MRP2 expressed in the apical membrane of the intestine epithelial cell are involved in the excretion of exogenous compounds (i.e., drugs and toxic chemicals) to protect the body from toxic anions [15,34,35]. At a transcriptional level, it has been reported that the MRP2 are regulated by pregnane X receptor (PXR), farnesoid X receptor (FXR), and constitutive androstane receptor (CAR) [36]. As the cellular mechanism of inducing MRP2 by GTCs is still unclear, these findings presented a new line of research and should be explored further.

Thirdly, all of the GTCs could significantly increase the protein level of BCRP, while only EC, CG, ECG, CGC, and EGCG showed an upregulation of the BCRP gene expression (Figure 9C). The results showed that the trans catechins had a better inductive effect on the mRNA levels of the BCRP than the cis (epi) catechins, except for C and EC, which indicated that the dimensional structure of the catechins could also produce a significant influence on the upregulation of BCRP at a transcription level. As for the protein level, no significant differences were found between the cis–trans catechins. The upregulation of BCRP caused by the EGCG treatment are inconsistent with a previous report, in which the mRNA transcription and protein level of BCRP were not changed after EGCG treatment (100 μg/mL) in the MCF-7 cell line (a breast cancer cell line eatablished by Michigan Cancer Foundation-7) [37]. The difference may be caused by the different cell lines from the different tissues.

## 3. Materials and Methods

### 3.1. Materials

Stock cultures of the Caco-2 cells (the TC7 clone-human adenocarcinoma) were obtained from the cell collection of the Chinese Academy of Sciences (Shanghai, China). Dulbecco’s modified eagle medium (DMEM), fetal bovine serum (FBS), nonessential amino acids, penicillin/streptomycin, Hanks’ Balanced Salts Solution (HBSS), phosphate buffered saline (PBS) tablets, and 0.05% trypsin–Ethylenediaminetetraacetic acid (EDTA)were purchased from HyClone Laboratories Inc. (Logan, UT, USA), and the plasmocin was purchased from InvivoGen (San Diego, CA, USA). (+)-catechin (C), (−)-epicatechin (EC), (−)-gallocatechin(GC), (−)-epigallocatechin (EGC), (−)-catechin gallate (CG), (−)-epicatechin gallate (ECG), (−)-gallocatechin gallate (GCG), (−)-epigallocatechin gallate (EGCG), and ethyl gallate at a purity of ≥98% were purchased from Shanghai Yuanye Biological Technology Company Limited (Shanghai, China). MK-571 sodium salt hydrate (MK571) at a purity of ≥95% were from Sigma-Aldrich Chemical Co. (St. Louis, MO, USA). Methanol, acetonitrile, and trifluoroacetic acid at HPLC grade were from Thermo Fisher Scientific (Waltham, MA, USA). All of the other reagents and solvents were in analytical grade or above, purchased from China National Pharmaceutical Group Corporation (Beijing, China). Ultrapure water (≥18.2 MΩ at 25 °C) was prepared using a Millipore Mill-Q Ultrapure Water System (Billerica, MA, USA). Rabbit BCRP antibody was purchased from Cell Signaling Technology (Danvers, MA, USA). Rabbit *P*-glycoprotein antibody, rabbit MRP2 antibody, mouse β-actin antibody, goat anti-rabbit, and goat anti-mouse secondary antibody were all from Proteintech Group (Wuhan, China).

### 3.2. Methods

#### 3.2.1. Cell Culture

The Caco-2 cells were cultured as described by Kelly Johnston [38]. Briefly, stock cultures of the Caco-2 cells were seeded in 25 cm^2^ plastic flasks, and were incubated in a 95% air/5% CO^2^ atmosphere in DMEM, supplemented with 10% fetal bovine serum, 1% penicillin/streptomycin, 1% non-essential amino acids, 1% L-glutamine, and 0.25 mg of plasmocin. The medium was replaced every two days and the cells were subculture at 80%–90% confluence by trypsiniztion with 0.05% trypsin–EDTA. All of the experiments were carried out on cells between passage numbers 32 and 47.

#### 3.2.2. Detection of Caco-2 Viability

The Cell Counting Kit-8 (CCK-8) assay was used to detect the viability of Caco-2 cells with different catechins. Briefly, the Caco-2 cells were seeded in 96 well plates (1.0 × 10^4^ cells/well) under culture condition for 48 h, with five replicates for each concentration. Then, the medium was removed and replaced with complete mediums each containing catechins at different concentrations (100 μmol/L to 600 μmol/L). After incubation for 24 h, the plate was gently washed twice with PBS and replaced with 100 μL complete mediums. Then, 10 μL CCK-8 reagents were added to each well, and they were incubated for an additional 1 h. The absorbance was measured at 450 nm using a spectrophotometer (722N, Shanghai Jinghua Science and Technology Instruments Co., Ltd., Shanghai, China), and the viability of the Caco-2 cells (%) was calculated according to the following equation:

Viability of Caco-2 cells (%) = (OD_sample_ − OD_blank_)/(OD_control_ − OD_blank_) × 100 (1)

where the OD_sample_ is the absorbance value of “cell + sample + complete medium + CCK-8”, OD_control_ is the absorbance value of “cell + complete medium + CCK-8”, and OD_blank_ is the absorbance value of “complete medium + CCK-8”.

#### 3.2.3. Transepithelial Transport Experiments across Caco-2 Monolayer

For the transport experiments, the cells were seeded in 24-mm i.d Transwell^®^ inserts (polycarbonate membrane, 0.4 μM pore size, Corning Costar Co., (Corning, NY, USA) in six-well plates at a density of 4 × 10^5^ cells/well, and were cultured for 21 days prior to the transport experiments. To monitor the integrity of the cell layers, transepithelial electrical resistance (TEER) values were measured using a Millicell-ERS Voltohmmeter (Millipore Corp, Bedford, MA, USA). TEER values above 513.7 Ω·cm^2^ in the culture medium were selected for the transport experiment.

For the transport of GTCs across the Caco-2 monolayers, the inserts were gently washed twice and were equilibrated at 37 °C for 30 min with 0.01 M modified phosphate buffer saline (PBS+), which was supplemented with 0.45 M calcium chloride and 0.4 M potassium chloride, and adjusted to pH 6.0 [7]. In the transepithelial transport, GTCs at different concentrations in PBS+ (DMSO less than 0.5%) were loaded into the apical (AP) or basolateral (BL) side, the so-called donor side. The plate was then incubated at 37 °C. At designated time intervals, aliquots of 0.5 mL of sample were taken from the other side, the so-called receiver side, and then replaced with an equal volume of PBS+. After that, the samples were dried with a CoolSafe freeze dryer (LaboGene ApS, Bjarkesvej, Denmark) at −110 °C, and stored at −20 °C until analysis.

For the inhibition of transport, MK571 was used at a concentration of 93.11 μmol/L (50 μg/mL with a DMSO less than 0.3%). The cells were preincubated with the inhibitor for 30 min. Then, the bi-directional transport studies of the GTCs in the presence of MK-571 at both the donor and receiver side were performed as for the transport method described above.

#### 3.2.4. HPLC Analysis

The transport samples were analyzed by an Agilent LC 1260 system (Agilent Technologies, CA, USA) after redissolving with 50 μL methyl into vial inserts (100 uL; Agilent Technologies, CA, USA). The separation was performed on an Octadecylsilyl (ODS) reversed-phase column (4.6 mm i.d. × 250 mm, 4.5 μm; TC-C18, Agilent Technologies) using a diode array detector (DAD) at 278 nm. The mobile phase consisted of 15% methanol and 0.1% formic acid at a flow-rate of 0.8 mL/min. The quantitation was done by peak area measurements in comparison with the standard curves for GTCs (as shown in Supplement 1).

#### 3.2.5. LC/MS Analysis

To analyze the metabolites of the tea catechins, samples taken from the transport studies were analyzed by reverse phase HPLC with MS detection, with ethyl gallate (20 μg/mL) as the internal standard [26]. Afterwards, separations were performed onUPLC-Q-TOF-MS/MS (Q-TOF 6520, Agilent Technologies, CA, USA) with an Eclipse XDB-C18 column (2.1 × 100mm, 1.8 μm; Agilent Technologies) at 35 °C. The HPLC mobile phase gradient began with a 10% mobile phase A (acetonitrile containing 0.1% trifluoroacetic acid) and a 90% mobile phase B (water containing 0.1% trifluoroacetic acid), and was changed linearly to 30% mobile phase A and 70% mobile phase B in 20 min. Then, the gradient was changed back to the original composition in 2 min. After separation, (−)-electrospray ionization (ESI in negative mode) was set for the analysis. The other mass spectrometer parameters were as follows: ESI capillary −3.5 kv, desolvation temperature 350 °C, source temperatures 300 °C, nebulization gas 35 psi, and flow rate 8 L/min. The possible metabolites of each tea catechins, including methylated conjugate, sulfate conjugate, glucuronidated conjugate, and methylated sulfate conjugate, were monitored by targeting their related deprotonated molecular ions.

#### 3.2.6. Quantitative Real Time RT-PCR

The Caco-2 cells were seeded in six-well plates (Corning Costar Co., NY, USA) at a density of 4 × 10^4^ cells/cm^2^, and were cultured for 21 days. After incubation with each tea catechins (300 μmol/L in full culture medium) for 2 h or 24 h, the total RNA from the Caco-2 cells were isolated with a Total RNA Kit (Aidlab, Beijing, China). First-strand cDNA was synthesized using a cDNA synthesis Kit (Aidlab, Beijing, China). The gene expression of MRP2 was examined by quantitative real-time PCR with the SYBR Green qPCR Mix (Aidlab, Beijing, China) in an ABI7500 Real-Time System (Applied Biosystems, USA). The reaction was carried out in a 10 μL RT-reaction mixture containing 5 μL 2 × SYBR Green qPCR Mix, 0.2 μL forward and reverse primers (10 μmol/μL), and water to a final volume of 10 μL, and was performed under the following cycling conditions: an initial denaturing step of 10 min at 95 °C, followed by 40 cycles consisting of 5 s at 95 °C and 34 s at 60 °C.

All of the qPCR experiments were repeated in three biologicals. The data were analyzed using the 2-ΔΔCT method [39]. All of the primers were synthesized by Sangon Biotech (Shanghai, China), as previously described [40], and their sequences are depicted in Table 3.

#### 3.2.7. Western Blot

After being washed by precooled PBS buffer three times, the total lysate was prepared on ice from 5 to 8 × 10^6^ cells by lysis in a 150 μL Radio Immunoprecipitation Assay (RIPA) Lysis buffer containing 1% (*v*/*v*) Phenylmethanesulfonyl fluoride (PMSF) (Beyotime, Shanghai, China). The lysates were centrifuged at 13,000 rpm for 20 min at 4 °C, after mixing uniformity. The protein-containing supernatant was collected and the protein content was determined using the Bio-Rad protein assay. Western blotting was performed according to standard techniques, as previously described [29]. In each lane of a Bio-Rad minigel system, 10 μg of protein was loaded. For the detection of BCRP, *P*-glycoprotein (MDR1), and MRP2, the following polyclonal antibodies were used: rabbit anti-BCRP (1:1000), rabbit anti-*P*-glycoprotein (1:1000), and rabbit anti-MRP2 (1:600). Horseradish peroxidase (HRP)-conjugated goat anti-rabbit (1:3000) were used as the secondary antibodies. As a loading control, the expression of β-actin was determined using an antibody against β-actin (1:40,000). Enhanced chemiluminescence (ECL) solutions were used for chemiluminescence.

#### 3.2.8. Data Analysis

The apparent permeability the coefficient (*P_app_*) and efflux ratio was calculated, as described previously [41]. All of the data analysis was performed using SPSS statistical software (SPSS, Chicago, IL, USA). The values were expressed as the mean ± standard deviation (SD) of three replications. The statistical significances of the difference between the various groups were analyzed using the Fisher’s least significant difference (LSD) procedure. For comparison between the two groups, the Student’s *t*-test was used. The differences were considered to be significant when *p* < 0.05.

## 4. Conclusions

Trans catechins (C, GC, CG, and GCG) showed limited transepithelial absorptions with low *P_app_* (all less than 1 × 10^−6^ cm/s) values, while a significant efflux inhibited by MK-571 was found during secretion. It suggested that catechins might be substrates of efflux transporters inhibited by MK-571, including MRP2, *P*-glycoprotein, and BCRP. By comparing the transport and metabolism of cis and trans catehichins, the results showed that catechin stereochemistry significantly affects the efflux transport rather than absorption transport in Caco-2 monolayers. The trans catechins showed a better transcellular permeability than their corresponding cis (epi) catechins in efflux transport at each concentration and time point tested. Moreover, more metabolites (quantitatively and qualitatively) were found after the transcellular efflux of trans catechins, when compared with their corresponding cis (epi) catechins, which suggested that more metabolites of trans catechins were fluxed into the lumen than the cis (epi) catechins after absorption in humans. These provide evidence that cis (epi) catechins may have a better oral bioavailability than trans catechins in humans. Furthermore, the results that the GTCs significantly increased the expression of MRP2 and BCRP, and decreased the expression of the *P*-glycoprotein in the Caco-2 cells have been found for the first time. The regulation of GTCs on P-glycoprotein, MRP2, and BCRP could also be significantly influenced by the dimensional structure. These findings presented a new line of research and should be explored further.

## Figures and Tables

**Table 1:** The *P_app_* values of the eight catechins with and without the treatment of MK-571 sodium salt hydrate (MK571). Each value was the average of three different samples; mean values with the same lowercase letters in the same column indicate no significant difference at a 0.05 level; * indicate *P_app_* values (apical (AP) to basolateral (BL)) in the absence of MK571 are significantly different from that (AP to BL) in the presence of MK571 (*p* < 0.05); ^#^ indicates *P_app_* values (BL to AP) in the absence of MK571 that are significantly different from that (BL to AP) in the presence of MK571 (*p* < 0.05). EC: (−)-epicatechin: C: (+)-catechin; EGC: (−)-epigallocatechin; GC: (+)-gallocatechin; ECG: (−)-epicatechin gallate; CG: (−)-catechin gallate; EGCG: (−)-epigallocatechin gallate; GCG: (−)-gallocatechin gallate.

	Transport in the Absence of MK571 (10^−7^ cm/s)		Efflux Ratio	Transport in the Presence of MK571 (10^−7^ cm/s)		Efflux Ratio
	AP to BL	BL to AP		AP to BL	BL to AP	
C	1.56 ± 0.11 ^b^	26.18 ± 1.52 ^a^	16.74	3.97 ± 0.55 ^a,b^,*	5.33 ± 0.45 ^a,#^	1.34
EC	1.52 ± 0.17 ^b^	16.94 ± 1.63 ^b^	11.16	4.39 ± 0.45 ^a,^*	4.65 ± 0.53 ^b,#^	1.06
CG	1.39 ± 0.12 ^b^	6.51 ± 0.42 ^d^	4.68	2.85 ± 0.10 ^c,^*	3.85 ± 0.22 ^c,#^	1.35
ECG	1.38 ± 0.09 ^b^	3.87 ± 0.31 ^e^	2.82	2.63 ± 0.22 ^c,^*	2.72 ± 0.30 ^d,#^	1.04
GC	2.64 ± 0.21 ^a^	8.75 ± 0.28 ^c^	3.32	3.43 ± 0.08 ^b,c,^*	4.45 ± 0.46 ^b,#^	1.30
EGC	2.46 ± 0.10 ^a^	6.74 ± 0.62 ^d^	2.74	3.63 ± 0.35 ^b,^*	3.67 ± 0.13 ^c,#^	1.01
GCG	1.03 ± 0.21 ^c^	2.93 ± 0.13 ^e^	2.84	1.73 ± 0.16 ^d,^*	2.14 ± 0.16 ^e,#^	1.24
EGCG	0.93 ± 0.08 ^c^	2.57 ± 0.10 ^e^	2.75	1.89 ± 0.18 ^d,^*	1.98 ± 0.20 ^e,#^	1.05

**Table 2:** Transition ions, retention times (for each conjugation position), and relative contents of green tea catechins (GTCs) metabolites from the samples taken from the apical sides after loading catechins in the basolateral side. The relative content of the catechin metabolites were measured as free-form equivalents, relative to the internal standard (ethyl gallate). Each value was the average of three different samples. * and ** indicate significantly different at a 0.05 and 0.01 level between the metabolites from cis and trans catechins, respectively. Me indicates methyl; ND indicates not detected.

Compound Name	Detected Mass (ESI-)	MS/MS Fragments (Collision Energy)	Retention Time (min)	Relative Content (nmol)	Significance
*C*-*O*-sulfate	369.0279	289, 231, 109 (20 V)	3.86	541.57 ± 65.43	*
EC-*O*-sulfate	369.0285	289, 245,137 (20 V)	6.69	202.50 ± 12.58	
*O*-me-*C*	303.0879	248, 203, 159, 101, 73 (1 0V)	3.86, 9.25	41.07 ± 3.66	*
*O*-me-EC	303.089	248, 159, 73 (10 V)	7.40, 12.64	22.33 ± 1.85	
*O*-me-*C*-*O*-sulfate	ND	ND	ND	ND	
*O*-me-EC-*O*-sulfate	ND	ND	ND	ND	
GC-*O*-sulfate	385.024	305, 261, 125 (20 V)	2.19	510.85 ± 61.72	**
EGC-*O*-sulfate	385.0246	305, 219, 154 (20 V)	2.54	31.77 ± 1.97	
*O*-me-GC	319.0523	303, 194, 105 (10 V)	2.17, 7.47	18.54 ± 1.74	*
*O*-me-EGC	319.0523	253, 183, 93 (10V)	2.35, 8.10	30.10 ± 3.35	
*O*-me-GC-*O*-sulfate	399.0395	385, 288, 124 (10 V)	2.20, 3.25	79.42 ± 6.50	*
*O*-me-EGC-*O*-sulfate	399.0404	319, 253, 183, 93 (10 V)	2.52, 4.23	23.14 ± 1.60	
CG-*O*-sulfate	521.0416	441, 369, 289, 249, 169, 64 (20 V)	13.61	366.00 ± 44.22	**
ECG-*O*-sulfate	521.0406	441, 331, 289, 169, 125 (20 V)	16.28	7.25 ± 0.45	
*O*-me-CG	455.0998	439, 382, 271, 169, 125, 58 (20 V)	14.69, 18.65, 19.08	50.78 ± 5.44	*
*O*-me-ECG	455.0989	352, 183, 109 (20 V)	14.13, 17.14	10.02 ± 0.80	
*O*-me-CG-*O*-sulfate	535.0567	455, 397, 303, 198, 141, 89 (10 V)	15.69, 17.11	62.89 ± 6.10	
*O*-me-ECG-*O*-sulfate	ND	ND	ND	ND	
GCG-*O*-sulfate	537.0362	457, 395, 305, 216, 169, 125 (10 V)	7.48, 8.79, 9.76	216.70 ± 20.73	**
EGCG-*O*-sulfate	537.0376	457, 287, 169, 113 (10 V)	10.4	6.87 ± 0.55	
*O*-me-GCG	471.0931	402, 287, 125 (10 V)	9.82, 14.61, 15.12	35.23 ± 3.67	*
*O*-me-EGCG	471.0954	305, 113 (10 V)	7.81, 12.19	11.51 ± 1.11	
*O*-me-GCG-*O*-sulfate	551.0513	464, 399, 369, 324, 205, 157, 77 (10 V)	11.84, 13.50, 14.68	96.48 ± 7.80	
*O*-me-EGCG-*O*-sulfate	ND	ND	ND	ND	

**Table 3:** Primer sequences for qPCR assays. MRP2: multidrug resistance-associated protein 2; BCRP: breast cancer-resistance protein

Gene	Forward Primer	Reverse Primer
*P*-glycoprotein/ABCB1	GCCAAAGCCAAAATATCAGAC	TTCCAATGTGTTCGGCAT
MRP2/ABCC2	TGAGCAAGTTTGAAACGCACAT	AGCTCTTCTCCTGCCGTCTCT
BCRP/ABCG2	TGCAACATGTACTGGCGAAGA	TCTTCCACAAGCCCCAGG
β-actin	CAAGATCATTGCTCCTCCTGA	AGTCCGCCTAGAAGCATTTG

## Supplementary Materials

The results of the CCK-8 assay are shown in Figure S1; The MS/MS (negative ion) spectra of metabolites treated with C, CG, GC, and GCG are shown in supplement Figures S2–S5, respectively; the standard curves of the GTCs are shown in Table S1.

## References

[1] Yamamoto T., Juneja L.R., Chu S.C. (1997). Chemistry and Applications of Green Tea.

[2] Huang Y., Xu R., Song B., Yang S., Zhao L., Wu S. (2009). Effects of (−)-Epigallocatechin gallate on some protein factors involved in the epidermal growth factor receptor signaling pathway. J. Nanjing Med. Univ..

[3] Huvaere K., Nielsen J.H., Bakman M., Hammershøj M., Skibsted L.H., Sørensen J., Vognsen L., Dalsgaard T.K. (2011). Antioxidant properties of green tea extract protect reduced fat soft cheese against oxidation induced by light exposure. J. Agric. Food Chem..

[4] Ju J., Lu G., Lambert J.D., Yang C.S. (2007). Inhibition of carcinogenesis by tea constituents. Semin. Cancer Biol..

[5] Masuda M., Suzui M., Lim J.T., Deguchi A., Soh J.W., Weinstein I.B. (2002). Epigallocatechin-3-gallate decreases VEGF production in head and neck and breast carcinoma cells by inhibiting EGFR-related pathways of signal transduction. J. Exp. Ther. Oncol..

[6] Wan S.B., Chen D., Ping Dou Q., Hang Chan T. (2004). Study of the green tea polyphenols catechin-3-gallate (CG) and epicatechin-3-gallate (ECG) as proteasome inhibitors. Bioorgan. Med. Chem..

[7] Zhang L., Zheng Y., Chow M.S.S., Zuo Z. (2004). Investigation of intestinal absorption and disposition of green tea catechins by Caco-2 monolayer model. Int. J. Pharm..

[8] Abrahamse S.L., Kloots W.J., van Amelsvoort J.M.M. (2005). Absorption, distribution, and secretion of epicatechin and quercetin in the rat. Nutr. Res..

[9] Zhu M., Chen Y., Li R.C. (2000). Oral absorption and bioavailability of tea catechins. Planta Med..

[10] Clifford M.N., van der Hooft J.J.J., Crozier A. (2013). Human studies on the absorption, distribution, metabolism, and excretion of tea polyphenols. Am. J. Clin. Nutr..

[11] Warden B.A., Smith L.S., Beecher G.R., Balentine D.A., Clevidence B.A. (2001). Catechins are bioavailable in men and women drinking black tea throughout the day. J Nutr.

[12] Kida K., Suzuki M., Matsumoto N., Nanjo F., Hara Y. (2000). Identification of biliary metabolites of (−)-epigallocatechin gallate in rats. J. Agric. Food Chem..

[13] Li C., Meng X., Winnik B., Lee M.J., Lu H., Sheng S., Buckley B., Yang C.S. (2001). Analysis of urinary metabolites of tea catechins by liquid chromatography/electrospray ionization mass spectrometry. Chem. Res. Toxicol..

[14] Ottaviani J.I., Borges G., Momma T.Y., Spencer J.P.E., Keen C.L., Crozier A., Schroeter H. (2016). The metabolome of [2-(14) C] (−)-epicatechin in humans: Implications for the assessment of efficacy, safety, and mechanisms of action of polyphenolic bioactives. Sci. Rep..

[15] Daood M., Tsai C., Ahdab-Barmada M., Watchko J.F. (2008). ABC transporter (P-gp/ABCB1, MRP1/ABCC1, BCRP/ABCG2) expression in the developing human CNS. Neuropediatrics.

[16] Sever R., Brugge J.S. (2012). Differential selectivity of efflux transporter inhibitors in Caco-2 and MDCK-MDR1 monolayers: A strategy to assess the interaction of a new chemical entity with P-gp, BCRP, and MRP2. J. Pharm. Sci..

[17] Hong J., Lambert J.D., Lee S.H., Sinko P.J., Yang C.S. (2003). Involvement of multidrug resistance-associated proteins in regulating cellular levels of (−)-epigallocatechin-3-gallate and its methyl metabolites. Biochem. Biophys. Res. Commun..

[18] Vaidyanathan J.B., Walle T. (2003). Cellular uptake and efflux of the tea flavonoid (−) Epicatechin-3-gallate in the human intestinal cell line Caco-2. J. Pharmacol. Exp. Ther..

[19] Meunier V., Bourrié M., Berger Y., Fabre G. (1995). The human intestinal epithelial cell line Caco-2; Pharmacological and pharmacokinetic applications. Cell Biol. Toxicol..

[20] Hirohashi T., Suzuki H., Chu X.Y., Tamai I., Tsuji A., Sugiyama Y. (2000). Function and expression of multidrug resistance-associated protein family in human colon adenocarcinoma cells (Caco-2). J. Pharmacol. Exp. Ther..

[21] Artursson P., Karlsson J. (1991). Correlation between oral drug absorption in humans and apparent drug permeability coefficients in human intestinal epithelial (Caco-2) cells. Biochem. Biophys. Res. Commun..

[22] Wan Y.F. (2006). Metabolism of green tea catechins: An overview. Curr. Drug Metab..

[23] Chen L., Lee M.J., Li H., Yang C.S. (1997). Absorption, distribution, elimination of tea polyphenols in rats. Drug Metab. Dispos..

[24] Sugihara N., Kuroda N., Watanabe F., Choshi T., Kamishikiryo J., Seo M. (2017). Effects of catechins and their related compounds on cellular accumulation and efflux transport of mitoxantrone in Caco-2 cell monolayers. J. Food Sci..

[25] Ottaviani J.I., Momma Y.T., Heiss C., Kwik-Uribe C., Schroeter H., Keen C. (2010). The stereochemical configuration of flavanols influences the level and metabolism of flavanols in humans and their biological activity in vivo. Free Radic. Biol. Med..

[26] Clarke K.A., Dew T.P., Watson R.E.B., Farrar M.D., Bennett S., Nicolaou A., Rhodes L.E., Williamson G. (2014). High performance liquid chromatography tandem mass spectrometry dual extraction method for identification of green tea catechin metabolites excreted in human urine. J. Chromatogr. B.

[27] Kuhnle G., Spencer J.P., Schroeter H., Shenoy B., Debnam E.S., Srai S.K., Rice-Evans C., Hahn U. (2000). Epicatechin and catechin are O-methylated and glucuronidated in the small intestine. Biochem. Biophys. Res. Commun..

[28] Vaidyanathan J.B., Walle T. (2002). Glucuronidation and sulfation of the tea flavonoid (-)-epicatechin by the human and rat enzymes. Drug Metab. Dispos..

[29] Hirsova P., Karlasova G., Dolezelova E., Cermanova J., Zagorova M., Kadova Z., Hroch M., Sispera L., Tomsik P., Lenicek M. (2013). Cholestatic effect of epigallocatechin gallate in rats is mediated via decreased expression of Mrp2. Toxicology.

[30] Baba S. (2001). In vivo comparison of the bioavailability of (+)-catechin, (−)-epicatechin and their mixture in orally administered rats. J. Nutr..

[31] Meng X., Lee M.J., Li C., Sheng S., Zhu N., Sang S., Ho C.T., Yang C.S. (2001). Formation and identification of 4′-O-methyl-(−)-epigallocatechin in humans. Drug Metab. Dispos..

[32] Lu H., Meng X., Yang C.S. (2003). Enzymology of methylation of tea catechins and inhibition of catechol-O-methyltransferase by (−)-epigallocatechin gallate. Drug Metab. Dispos..

[33] Kitagawa S., Nabekura T., Kamiyama S. (2004). Inhibition of P-glycoprotein function by tea catechins in KB-C2 cells. J. Pharm. Pharmacol..

[34] Gerk P.M., Vore M. (2002). Regulation of expression of the multidrug resistance-associated protein 2 (MRP2) and its role in drug disposition. J. Pharmacol. Exp. Ther..

[35] Keppler D., König J. (2000). Hepatic secretion of conjugated drugs and endogenous substances. Semin. Liver Dis..

[36] Kast H.R., Goodwin B., Tarr P.T., Jones S.A., Anisfeld A.M., Stoltz C.M., Tontonoz P., Kliewer S., Willson T.M., Edwards P.A. (2002). Regulation of multidrug resistance-associated protein 2 (ABCC2) by the nuclear receptors pregnane X receptor, farnesoid X-activated receptor, and constitutive androstane receptor. J. Biol. Chem..

[37] Farabegoli F., Papi A., Bartolini G., Ostan R., Orlandi M. (2010). (−)-Epigallocatechin-3-gallate downregulates Pg-P and BCRP in a tamoxifen resistant MCF-7 cell line. Phytomedicine.

[38] Johnston K., Sharp P., Clifford M., Morgan L. (2005). Dietary polyphenols decrease glucose uptake by human intestinal Caco-2 cells. FEBS Lett..

[39] Livak K.J., Schmittgen T.D. (2001). Analysis of relative gene expression data using real-time quantitative PCR and the 2(-Delta Delta C(T)) Method. Methods.

[40] Maubon N., Le Vee M., Fossati L., Audry M., Le Ferrec E., Bolze S., Fardel O. (2007). Analysis of drug transporter expression in human intestinal Caco-2 cells by real-time PCR. Fundam. Clin. Pharm..

[41] Vaidyanathan J.B., Walle T. (2001). Transport and metabolism of the tea flavonoid (−)-epicatechin by the human intestinal cell line Caco-2. Pharm. Res..

