# Supplementary material for: Effect of Stereochemical Configuration on the Transport and Metabolism of Catechins from Green Tea across Caco-2 Monolayers
Source: Molecules. 2019 Mar 26;24(6):1185. doi: 10.3390/molecules24061185 (PMC6470784; doi:10.3390/molecules24061185)
Supplement: Supplementary file 1 [file molecules-24-01185-s001.pdf]

Figure S1**. The viability of Caco-2 cell with different concentrations of GTCs: (A) C and EC; (B) GC and EGC; (C) CG and ECG; (D) GCG and EGCG**

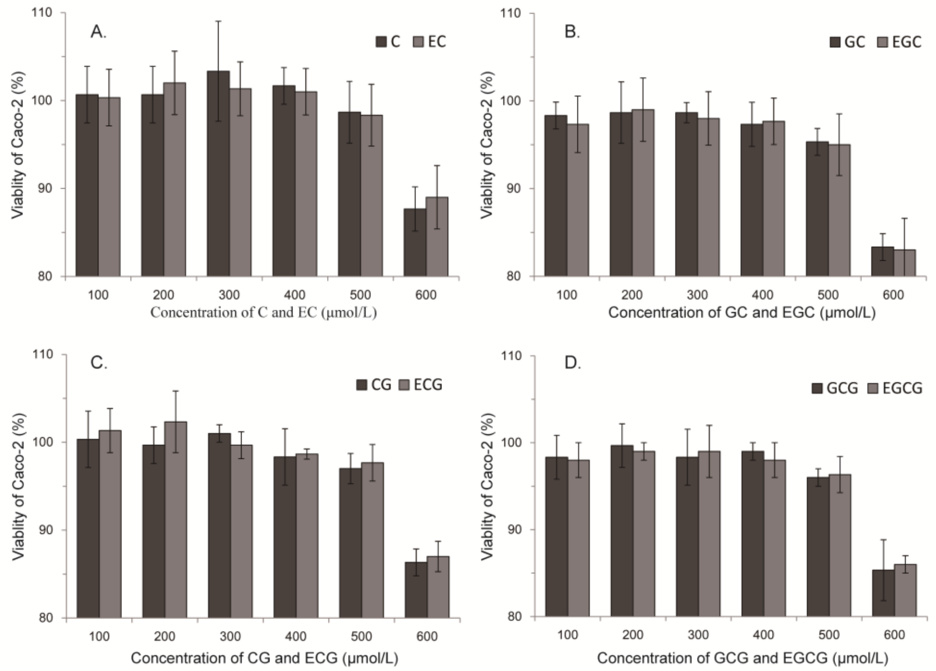

Figure S2**: MS/MS (negative ion) spectra of C metabolites**

The MS/MS spectra were corresponding to (A) m/z 369 [M-H]$^-$ and (B) 303 [M-H]$^-$ in the samples taken from apical sides after loading C in basolateral side

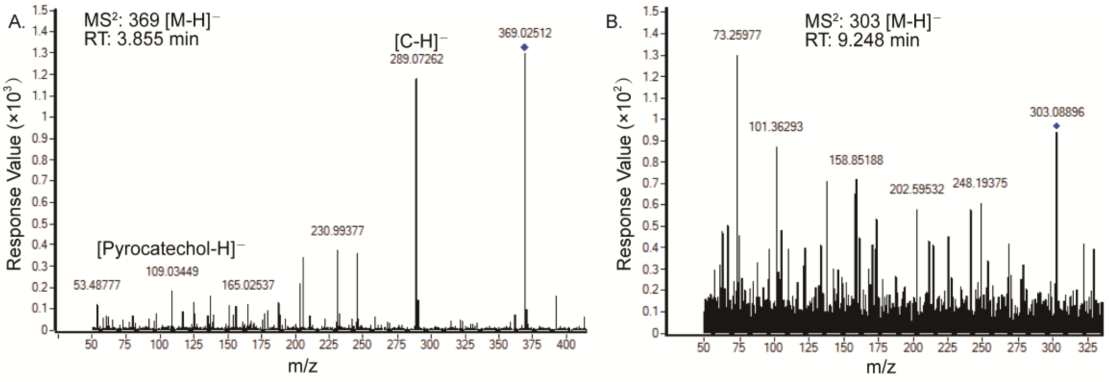

Figure S3**: MS/MS (negative ion) spectra of CG metabolites**

The MS/MS spectra were corresponding to (A) m/z 521 $[M-H]^-$, (B) m/z 455 $[M-H]^-$, (C) m/z 535 $[M-H]^-$ (15.694 min) and (D) m/z 535 $[M-H]^-$ (17.110 min) in the samples taken from apical sides after loading CG in basolateral side

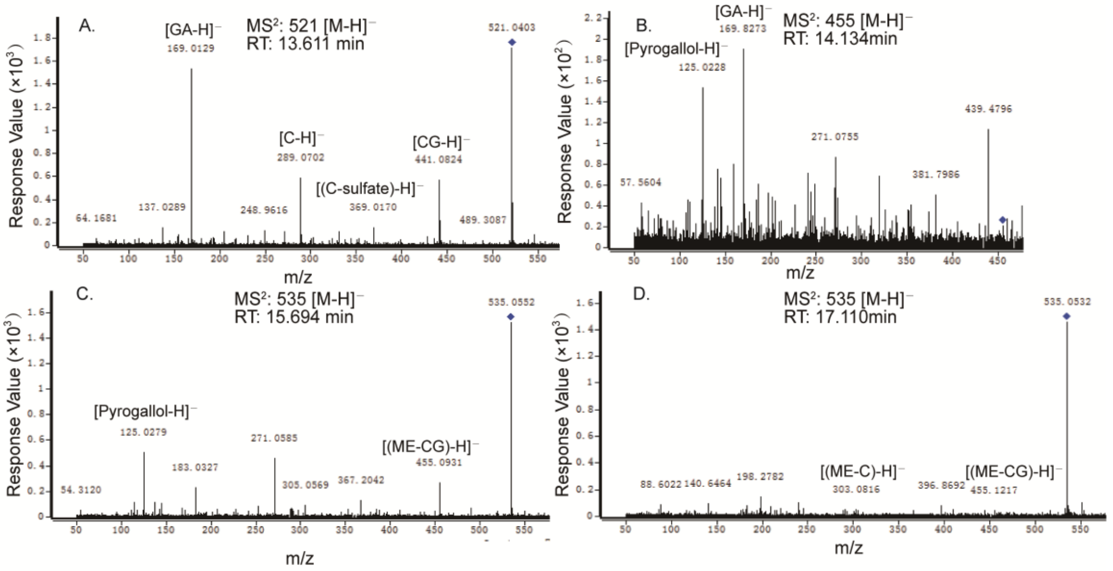

Figure S4**: MS/MS (negative ion) spectra of GC metabolites**

The MS/MS spectra were corresponding to (A) m/z 385 $[M-H]^-$ and (B) m/z 399 $[M-H]^-$ in the samples taken from apical sides after loading GC in basolateral side

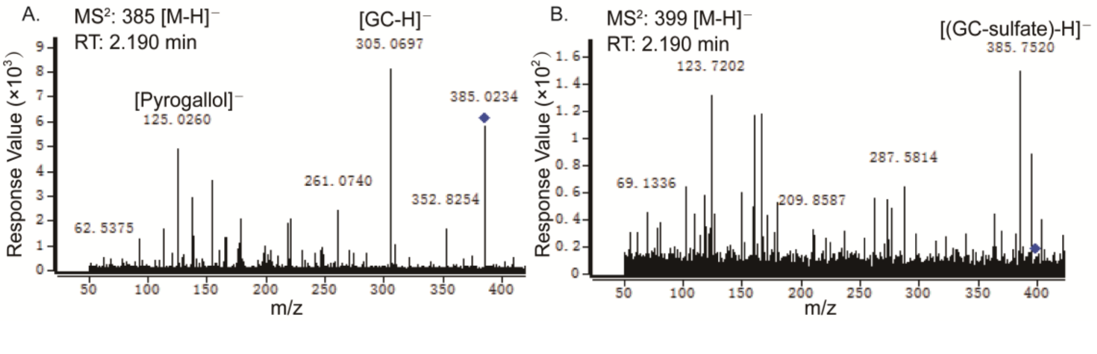

Figure S5**: MS/MS (negative ion) spectra of GCG metabolites**

The MS/MS spectra were corresponding to (A) m/z 537 [M-H]$^-$, (B) m/z 471[M-H]$^-$ and (C) m/z 551[M-H]$^-$ in the samples taken from apical sides after loading GCG in basolateral side

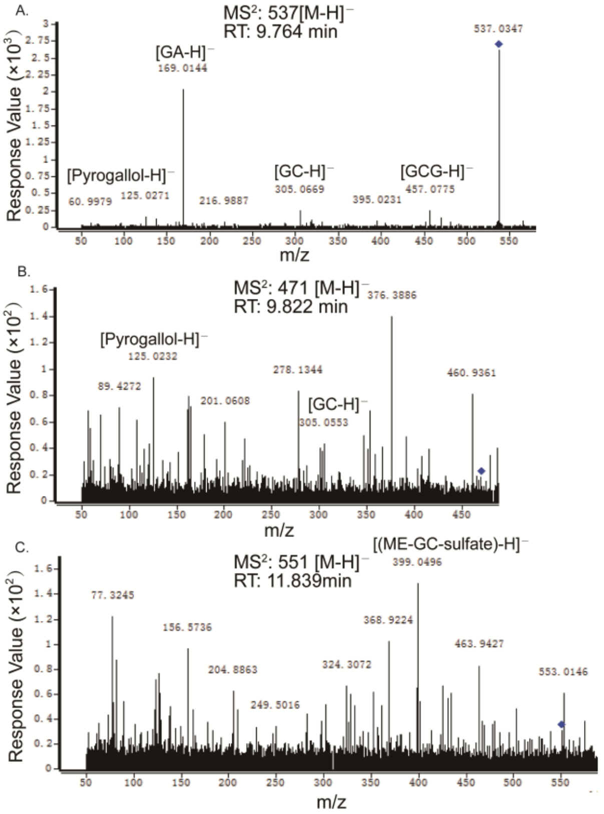

Table S1**. Standard curves of GTCs**

Catechins	Regression equation	Correlation coefficient	Linear range（μg/mL）
C	y=4.6655x + 0.1765	0.9997	1.0-100.0
EC	y=4.6512x + 5.0886	0.9990	2.0-100.0
CG	y=15.966x + 5.5271	0.9990	1.0-100.0
ECG	y=11.121x-8.4906	0.9998	0.5-100.0
GC	y = 1.3481x - 2.5387	0.9996	0.2-100.0
EGC	y=6.343x - 25.293	0.9995	2.0-100.0
GCG	y=8.8984x - 9.9642	0.9998	1.0-100.0
EGCG	y=8.2688x - 13.049	0.9997	2.0-100.0
